# Effectiveness of galcanezumab on sleep quality, migraine outcome, and multidimensional patient-reported outcome measures: a real-world experience in Turkish patients with episodic and chronic migraine

**DOI:** 10.3389/fneur.2024.1411238

**Published:** 2024-06-03

**Authors:** Elif Ilgaz Aydinlar, Tuba Erdogan Soyukibar, Pinar Yalinay Dikmen

**Affiliations:** Department of Neurology, Acibadem University School of Medicine, Istanbul, Türkiye

**Keywords:** migraine, galcanezumab, sleep quality, migraine outcome, MIDAS, HIT-6, quality of life, anxiety-depression

## Abstract

**Introduction:**

This real-world study aimed to investigate the impact of galcanezumab on sleep quality, migraine outcome and multidimensional patient-reported outcomes measures (PROMs) in patients with episodic migraine (EM) and chronic migraine (CM).

**Methods:**

Fifty-four patients with episodic migraine (*n* = 24) or chronic migraine (*n* = 30) received a 3-month series of galcanezumab injections and were evaluated for sleep quality, measured using the Pittsburgh Sleep Quality Index (PSQI), as well as migraine outcomes such as monthly headache days (MHDs), monthly migraine days (MMDs), and headache severity. Patient-reported outcome measures (PROMs) such as the Migraine Disability Assessment Scale (MIDAS), Headache Impact Test-6 (HIT-6), SF-36 Health-related Quality of Life (HRQoL), Beck Anxiety Inventory (BAI), and Beck Depression Inventory (BDI) were additionally included in the assessment.

**Results:**

The percentage of patients with poor sleep quality (total PSQI scores ≥ 5) was 72.7% at baseline, decreasing to 57.5% and 56.2% at the 1st and 2nd months, respectively. By the 3rd month of galcanezumab injections, significant improvement was observed in the sleep disturbances domain in the overall study population (*p* = 0.016), and in subgroups of patients with low anxiety levels (*p* = 0.016) and none/minimal depression (*p* = 0.035) at baseline. Patients with sleep disorder at baseline exhibited marked improvements in total PSQI scores (*p* = 0.027) and in the subjective sleep quality (*p* = 0.034) and daytime dysfunction (*p* = 0.013) domains, by the 3rd month. Over the 1st, 2nd, and 3rd months, there were significant improvements in MHDs (*p* < 0.001), MMDs (*p* < 0.001), HIT-6 scores (*p* < 0.001 for each), BAI scores (*p* < 0.001 for each), BDI scores (*p* ranged from 0.048 to <0.001), and HRQoL scores (*p* ranged from 0.012 to <0.001).

**Conclusion:**

Galcanezumab demonstrates notable benefits in improving sleep quality, along with a comorbidity-based and domain-specific effect on sleep parameters, which involved sleep disturbances domain in patients without depression or anxiety at baseline but the total PSQI scores, subjective sleep quality and daytime dysfunction in those with sleep disorder at baseline. The treatment also facilitates rapid-onset enhancements in migraine outcomes as well as various PROMs.

## 1 Introduction

Migraine is a highly prevalent disabling complex neurological disorder and a major cause of global population ill health with adverse effects on multiple domains (personal, psychosocial and economic) besides the neurobiological symptoms (1–3). Migraine remains inadequately treated despite its association with considerable disability, impaired functioning, and decreased quality of life (QoL) in patients and a substantial socioeconomic burden (2–5).

Many sleep disorders (i.e., insomnia, restless legs syndrome, sleep apnea, and daytime sleepiness) and psychiatric comorbidities (anxiety and depression in particular) are more prevalent among migraine patients than in the general population, as associated with increased headache frequency and migraine disability, poor treatment response and an increased risk for migraine progression to chronic form over time in these patients (4, 6–8).

The relationship between sleep and migraine is intricate (9). While their bidirectional comorbidity is well-established, the nature of this relationship is still not fully understood (4, 8). Patients with chronic migraine (CM) tend to experience more symptoms of insomnia compared to patients with episodic migraine (EM), suggesting a potential link between sleep disturbances and the progression of migraine (8, 10). Migraineurs are significantly more likely to suffer from poor sleep quality, insomnia and night-time fatigue (4). The frequency of headaches in CM patients decreased following insomnia treatment, highlighting the impact of sleep on migraine patterns (11). In addition, another study identified a correlation between high monthly headache frequency and diminished sleep quality within a broader population of migraine patients (12). This correlation emphasizes the complex connection between the frequency of headaches and the overall quality of sleep in individuals with migraine. Understanding and addressing these links could potentially lead to more effective strategies for managing and treating migraine in affected populations. A meta-analysis revealed that patients with migraine generally scored higher on the Pittsburgh Sleep Quality Index (PSQI) compared to healthy controls (13). Elevated PSQI scores, indicate poor sleep quality, and are associated with increased migraine-related burden (14).

Galcanezumab, a humanized monoclonal antibody (mAb) targeting calcitonin gene-related peptide (CGRP)-mediated signaling cascade, is specifically developed for migraine prophylaxis in adults with EM and CM (15, 16). Several studies have consistently shown the efficacy of galcanezumab in enhancing migraine outcomes, including improvements in functional and disability scores, all while maintaining a favorable safety and tolerability profile (15–17).

Indeed, most comorbidities are considered among the exclusion criteria in the clinical trial settings. Therefore, real-world galcanezumab studies in migraineurs with comorbidities such as sleep disorders and anxiety and/or depression are of critical importance to investigate the effectiveness of this treatment on different migraine facets beyond the reduction of both monthly headache days (MHDs) and monthly migraine days (MMDs) (16, 18). Studies on how galcanezumab treatment affects sleep in migraine patients are lacking. Our real-world study in EM and CM patients aimed to investigate the impact of the galcanezumab injection series on sleep quality and migraine outcome in addition to several multidimensional patient-reported outcome measures (PROMs) such as negative emotional states, Health-related Quality of Life (HRQoL), headache impact and migraine-related disability.

## 2 Materials and methods

### 2.1 Study population

In this retrospective cohort study, patients between 18 and 65 diagnosed with EM and CM were included, according to the third edition of the International Classification of Headache Disorders (ICHD-3) (19). Participants were recruited from a tertiary headache center and evaluated by experienced headache specialists. The physician documented the patients’ detailed sociodemographic data during their baseline interview and asked them to complete PROMs. The patients were seen again at the second, third, and fourth visits, 1 month apart. At each visit, changes in headache days and migraine characteristics were recorded according patients’ headache diaries, and they were asked to fill out PROMs. Patients who did not attend regular follow-ups or incompletely filled out the forms were not included.

During the study period, the enrolled patients did not receive any other prophylactic treatment for migraine, additional antidepressant therapy, sleep medications, nerve blocks, or trigger point injections. Exclusion criteria included pregnant or breastfeeding women, illiteracy, unstable medical conditions, as well as individuals who had recently initiated a new psychiatric medication or undergone dose adjustments for ongoing psychiatric medication within the 3 months preceding the study enrollment.

This study was approved by Acibadem University School of Medicine Medical Research Ethics Committee (Approval number: 2023-20/671).

### 2.2 Study parameters

Data on the participants’ demographic features, migraine type and comorbid diseases were documented at baseline. The sleep quality was evaluated with PSQI. The migraine outcomes were assessed using patient-recorded monthly headache days (MHDs), monthly migraine days (MMDs) and headache severity documented in a headache diary. PROMs including Migraine Disability Assessment Scale (MIDAS), Headache Impact Test-6 (HIT-6), 12-item Allodynia Symptom Checklist (ASC-12), SF-36 Health-related Quality of Life (SF-36 HRQoL), Beck Anxiety Inventory (BAI) and Beck Depression Inventory (BDI) were recorded at baseline and follow-up visits. A numeric rating scale (NRS) was used to describe pain severity (0 means no pain, 10 means the worst pain imaginable). Additionally, safety outcomes were evaluated during follow-up visits.

### 2.3 Galcanezumab injection series

Galcanezumab (Emgality®, Eli Lilly and Company, Indianapolis, United States; 120 mg/mL solution in a single-dose prefilled syringe) was administered subcutaneously in 240-mg loading dose (2 consecutive 120-mg injections) at baseline visit (visit 1), and then at 120 mg dose on a monthly basis for three consecutive visits including visit 2 (1st month), visit 3 (2nd month), and visit 4 (3rd month).

### 2.4 Responder definition

Galcanezumab responders were identified as individuals who experienced a reduction of 50% or more in monthly headache days (MHDs) between baseline and the third month of treatment. Conversely, non-responders were characterized as those with less than a 50% reduction in MHDs during the 3-month treatment period.

### 2.5 PSQI

The PSQI is a comprehensive 19-item questionnaire designed to evaluate sleep quality. It encompasses seven components, each contributing to a specific aspect of sleep assessment. These components include subjective sleep quality, sleep latency, sleep duration, habitual sleep efficiency, sleep disturbances, use of sleep medications, and daytime dysfunction. Each component is assigned a score ranging from 0 to 3, and the cumulative sum of these component scores produces a global score with a potential range of 0 to 21. Higher global scores indicate lower sleep quality. Interpreting the results, a total PSQI score below 5 is indicative of ‘good sleep quality’, while a score of 5 or higher suggests ‘poor sleep quality’. This scoring system provides a quantitative measure to assess and categorize an individual’s sleep patterns based on various components (20, 21).

### 2.6 MIDAS

The MIDAS is a self-administered questionnaire consisting of five items. Its purpose is to quantitatively assess headache-related disability by considering the number of days affected and the resulting activity limitations due to migraine over the past 3 months. The final total score is categorized depending on the severity of attacks as little or no disability (scores 0–5), mild disability (scores 6–10), moderate disability (scores 11 to 20) or severe disability (scores ≥ 21) (22, 23).

### 2.7 HIT-6

HIT-6 is a 6-item questionnaire with domains on pain, social functioning, role functioning, vitality, cognitive functioning, and psychological distress. Each item is answered on a 5-point Likert scale (6 = never, 8 = rarely, 10 = sometimes, 11 = very often, 13 = always). The total score ranges between 36 and 78 with higher scores reflecting more significant impact (24, 25).

### 2.8 SF-36 HRQoL

The SF-36 is a self-administered questionnaire designed to assess HRQoL across eight domains. These domains encompass physical functioning, physical and emotional role limitations, bodily pain, general health perception, vitality, social functioning, and mental health. The total scores derived from the SF-36 range from 0 to 100, with higher transformed scores serving as an indicator of a better health status (26, 27).

### 2.9 BDI

BDI is a 21-item self-reporting questionnaire for the assessment of the level and change in the severity of depression over the past 2 weeks, based on physical, emotional, cognitive, and motivational symptoms. Each item is scored on a 4-point scale from 0 (no symptom) to 3 (severe symptoms), while the total score (range, 0 to 63) is calculated by finding the sum of the 21 items with higher scores indicating greater symptom severity. Based on the total score individuals are categorized to have severe (scores 30–63), moderate (scores 19–29), mild (scores 10–18) and none/minimal depression (scores 0–9) (28, 29).

### 2.10 BAI

This 21-item scale is a self-report measure of anxiety. Each item is scored on a 4-point scale from 0 (not at all) to 3 (severely—it bothered me a lot), and the total score is calculated by finding the sum of the 21 items and classified as low (scores 0–21), moderate (scores 22–35) and potentially concerning levels of anxiety (scores ≥ 36) (30, 31).

### 2.11 Safety outcome

The assessment of safety outcomes in this context was conducted by considering various factors, including treatment-emergent adverse events (TEAE), serious adverse events (SAE), deaths, discontinuation rates, and monitoring vital signs such as blood pressure, pulse, temperature, and weight.

### 2.12 Statistical analysis

Statistical analysis was performed using the MedCalc® Statistical Software version 19.7.2 (MedCalc Software Ltd., Ostend, Belgium; https://www.medcalc.org; 2021). Shapiro–Wilk’s test investigated the normality of continuous variables. For comparison of more than two groups non-normally distributed continuous data Friedman Test was used. For *post hoc* evaluation, Bonferroni corrected Wilcoxon Signed Rank test was performed. No specific procedure was defined for missing data. Data were expressed as mean ± standard deviation (SD), median (inter-quartile range, IQR) and n (%), where appropriate. *p* < 0.05 was considered statistically significant.

## 3 Results

### 3.1 Patient demographics and comorbidities

Of the 85 patients who began galcanezumab treatment, 54 fully met the study criteria by completing the series of galcanezumab injections over 3 months, filling out PROMs without missing data, and were included in statistical analysis. Within the EM group, 24 patients, were categorized into high-frequency EM (HFEM) with 9–14 headache days per month for 12 patients, and low-frequency EM (LFEM) with 4–8 headache days per month for the remaining 12 patients. The CM group comprised 30 patients who experienced 15 or more headache days per month, with at least 8 days meeting the criteria for migraine with or without aura.

The mean age of the patients was 38.3 years (SD 10.1, range 33.5 to 44.0 years), with females constituting 90.7% (*n* = 49) of the study population. Most of patients were university graduates (77.8%) and employed (74.1%), while a family history for migraine was evident in 68.5% of patients Notably, 46.7% (*n* = 14) of the patients with CM also presented with medication overuse headache (MOH). Furthermore, comorbidities were identified, with 40.4% (*n* = 21) of patients having a sleep disorder, 36.5% (*n* = 19) having a psychiatric disease, and 26.9% (*n* = 14) having a gastrointestinal disease (Table 1).

**Table 1:** Baseline patient characteristics and migraine history (*n* = 54).

Age (year), mean(SD)	38.3(10.1)
**Gender, *n*(%)**	
Female	49(90.7)
Male	5(9.3)
**Educational status, *n*(%)**	
Primary school	2(3.7)
High school	10(18.5)
University	42(77.8)
**Employment, *n*(%)**	
Unemployed	14(25.9)
Employed	40(74.1)
**Family history for migraine, n(%)**	37(68.5)
**Body mass index (kg/m^2^), mean(SD)**	22.9(3.9)
**Comorbid diseases, *n*(%)**	
Sleep disorder	21(40.4)
Psychiatric disease	19(36.5)
Gastric disease	14(26.9)
**Type of migraine, *n*(%)**	
Chronic migraine	30(55.6)
Episodic migraine	24(44.4)
*HFEM*	12(22.2)
*LFEM*	12(22.2)
**Duration of migraine (years), mean(SD)**	16.4(9.5)
**Analgesic use, days, mean(SD)**	
Migraine non-specific	10.4(10.5)
Migraine specific	6.5(6.8)
**Previous treatments, *n*(%)**	
OnabotulinumtoxinA	13(24.1)
Antiepileptics	8(14.8)
Nerve blocks	7(13)
SSRI, SNRI	5(9.3)
CGRP monoclonal antibodies	2(3.7)
Other	4(7.4)

LFEM, Low-frequency episodic migraine; HFEM, High-frequency episodic migraine; SSRI, Selective serotonin re-uptake inhibitors; SNRI, Serotonin-norepinephrine reuptake inhibitors; CGRP, Calcitonin gene-related peptide.

### 3.2 Sleep quality

The total PSQI scores for all patients were as follows: median (IQR) 6 (4–11) at baseline. In the subgroups, LFEM had a median (IQR) score of 4.5 (3.7–10), HFEM had a median (IQR) score of 5 (4–6), and CM had a median (IQR) score of 8.5 (6–11.7) at baseline. The percentage of patients with poor sleep quality (total PSQI scores ≥ 5) was 72.7% at baseline, decreasing to 57.5% and 56.2% at the 1st and 2nd months, respectively (data not shown).

By the 3^rd^ month of galcanezumab injections, significant improvement was observed in the sleep disturbances domain in the overall study population (*p* = 0.016), and in subgroups of patients with low anxiety levels (p = 0.016) and none/minimal depression (*p* = 0.035) at baseline (Table 2).

**Table 2:** Sleep quality-PSQI scores.

PSQI scores, median (IQR)	Baseline	3rd month	*p*-value
**All patients (*n* = 54)**			
Total score	6(4.5–13)	5(3–9)	0.106
Subjective sleep quality	1(1–2)	1(1–2)	0.182
Sleep latency	2(1–2)	2(1–2)	0.898
Sleep duration	1(0–1)	1(0–1)	0.643
Habitual sleep efficiency	0(0–1)	0(0–0)	0.358
Sleep disturbances	2 (1–2)	1(1–2)	**0.016**
Use of sleep medications	2(0–2.3)	0(0–1.3)	0.260
Daytime dysfunction	1(0–2)	0(0–2)	0.108
**Patients with sleep disorder at baseline (*n* = 21)**			
Total score	8.5(4.3–14.8)	7.5(3.5–9.8)	**0.027**
Subjective sleep quality	2 (1–2.3)	1.5(1–2)	**0.034**
Sleep latency	2(1–3)	2(1–3)	0.084
Sleep duration	0(0–1.5)	1(0–1.5)	0.380
Habitual sleep efficiency	0(0–1)	0(0–0.1)	1.00
Sleep disturbances	2(1–2)	1(1–2)	0.059
Use of sleep medications	2(0–3)	0.5(0–3)	0.131
Daytime dysfunction	1.5(1–3)	1.5(0–2.3)	**0.013**
**Patients with low BAI scores at baseline (*n* = 40)**			
Total score	6(4–9.5)	5(3–8.5)	0.106
Subjective sleep quality	1(1–2)	1(1–2)	0.182
Sleep latency	2(1–2)	2(1–2)	0.898
Sleep duration	1(0–1.5)	1(0–1)	0.643
Habitual sleep efficiency	0(0–0)	0(0–1)	0.358
Sleep disturbances	2(1–2)	1(1–1)	**0.016**
Use of sleep medications	0(0–0.5)	0(0–0.3)	0.260
Daytime dysfunction	1(0–2)	0(0–2)	0.108
**Patients with none/minimal BDI scores at baseline (*n* = 24)**			
Total score	5(4–9)	4.5(3–7.3)	0.093
Subjective sleep quality	1(1–2)	1(1–1)	0.083
Sleep latency	1(1–2)	2(1–2)	0.480
Sleep duration	0.5(0–1)	1(0–1)	1.00
Habitual sleep efficiency	0(0–1)	0(0–0)	0.829
Sleep disturbances	1(1–2)	1(1–1)	**0.035**
Use of sleep medications	0(0–0)	0(0–0)	0.564
Daytime dysfunction	1(0–1)	0(0–1)	0.090

PSQI, Pittsburgh Sleep Quality Index; BAI, Beck Anxiety Inventory; BDI, Beck Depression Inventory. Friedman Test. Values in bold indicate statistical significance (*p* < 0.05).

Patients with sleep disorder at baseline exhibited marked improvements in total PSQI scores (*p* = 0.027) and in the subjective sleep quality (*p* = 0.034) and daytime dysfunction (*p* = 0.013) domains by the 3rd month (Table 2).

Moreover, there was no important difference documented between the chronic migraine (CM) and episodic migraine (EM) groups (total, LFEM, and HFEM) concerning the change from baseline to the 3^rd^ month in PSQI total and domain scores (Table 3).

**Table 3:** Changes in PSQI scores according to migraine type with galcanezumab treatment.

Change from baseline to 3rd month	Chronic migraine (CM)	Episodic migraine			*p*-value	
		All patients (A)	LFEM (B)	HFEM (C)	CM vs. A^1^	CM vs. B and C^2^
	(*n* = 30)	(*n* = 54)	(*n* = 12)	(*n* = 12)		
**PSQI scores**						
Total score	−1(−4–2)	0(−2–1.5)	−1(−5.3–1)	0.5(−1.8–3.8)	0.271	0.191
Subjective sleep quality	0(−1–0)	0(0–0)	0(−5–0)	0(0–5)	0.402	0.420
Sleep latency	0(−0.8–1)	0(0–0)	0(−5–0)	0(0–0)	0.571	0.777
Sleep duration	0(−1–0)	0(0–1)	0(0–1)	0(0–0.8)	0.090	0.186
Habitual sleep efficiency	0(−1–0)	0(0–0)	0(0–0)	0(0–0)	0.305	0.367
Sleep disturbances	0(−1–0)	0(−1–0)	−0.5(−1.3–0)	0(−0.5–0)	0.849	0.380
Use of sleep medications	0(0–0)	0(0–0)	0(0–0)	0(0–0)	0.471	0.771
Daytime dysfunction	0(−1–0)	0(−1–0)	0(−1–0)	0(−0.5–1)	0.617	0.652

*n*, Number of subject; PSQI, Pittsburgh Sleep Quality Index; LFEM, Low-frequency episodic migraine; HFEM, High-frequency episodic migraine. ^1^Mann-Whitney U test, ^2^Kruskal Wallis test.

### 3.3 Migraine outcome

**Figure 1:**
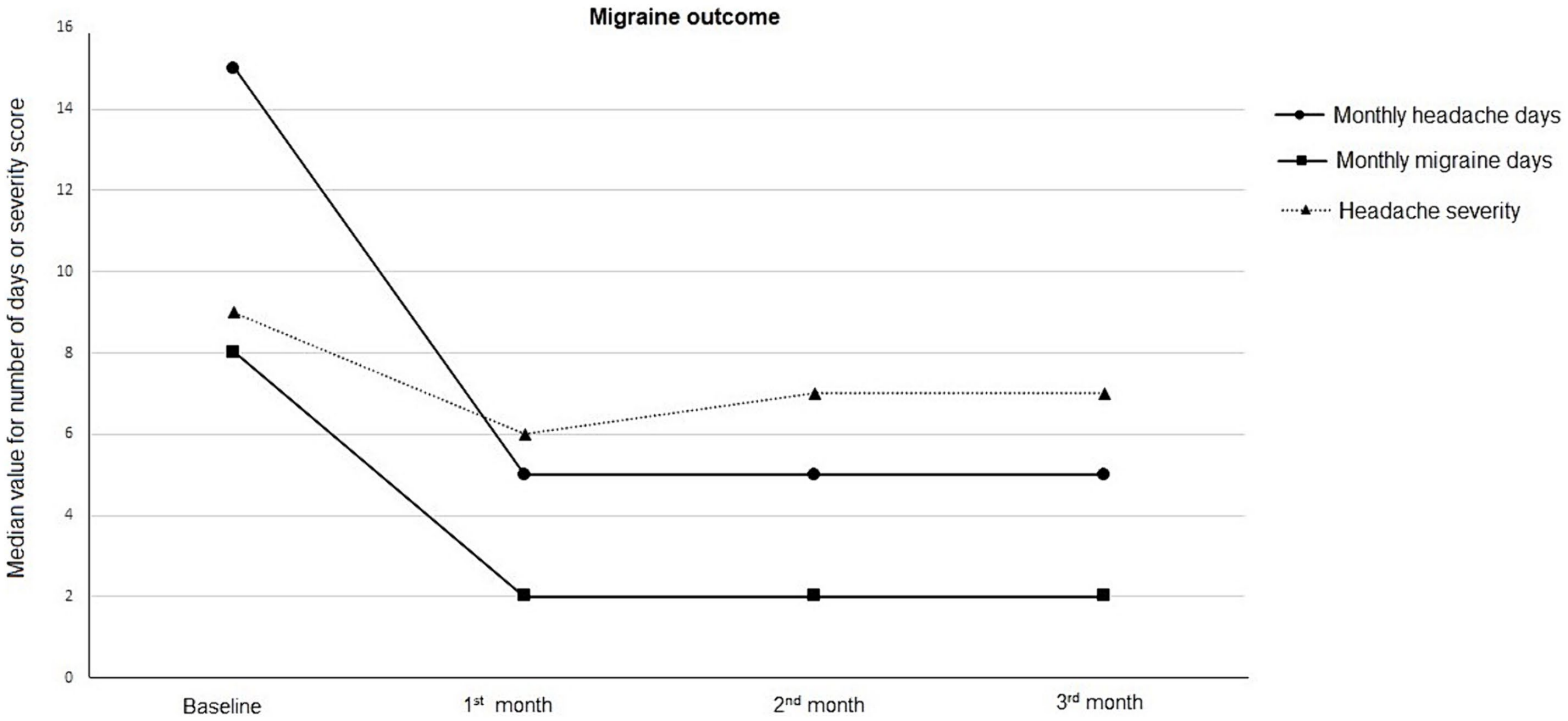
Migraine outcome in terms of monthly headache days, monthly migraine days, and headache severity, at baseline and follow-up visits.

Galcanezumab demonstrated marked improvement in migraine outcomes from baseline to the 1st, 2nd, and 3rd months, as indicated by the median (IQR) values:

– Monthly Headache Days: Decreased from 15 (9–22.5) days at baseline to 5 (3–10) days, 5 (2–7) days, and 5 (3–9.5) days at the 1st, 2nd, and 3rd months, respectively (*p* < 0.001 for each).
– Monthly Migraine Days: Reduced from 8 (5–10) days at baseline to 2 (1–5) days, 2 (1-4) days, and 2 (1–5) days at the 1st, 2nd, and 3rd months, respectively (*p* < 0.001 for each).
– Numeric Rating Scale: Decreased from 9 (8-9) at baseline to 6 (5–7), 7 (5–8), and 7 (5–8) at the 1st, 2nd, and 3rd months, respectively (*p* < 0.001 for each) (Figure 1).

### 3.4 Headache impact and migraine-related disability

HIT-6 scores exhibited a baseline value of 67 (65–70) and significantly decreased to 58 (50–62), 57 (48–62), and 56 (49–63) at the 1st, 2nd, and 3rd month visits, respectively (*p* < 0.001 for each). Additionally, MIDAS scores demonstrated marked improvement from baseline to the 3rd month, decreasing from 50 (0–180) to 9 (0–70) (*p* < 0.001) (Table 4).

**Table 4:** Patient reported outcome measures on headache impact and migraine-related disability, quality of life and anxiety-depression scores with galcanezumab treatment.

PROMs, median (IQR) (*n* = 54)	Baseline (A)	1st month (B)	2nd month (C)	3rd month (D)		*p*-value^1^
**MIDAS score**	50(0–180)	-	-	9(0–70)		**<0.0012**
**HIT-6 score**	67(65–70)	58(50–62)	57(48–62)	56(49–63)		**<0.001**
**SF36 HRQoL, median (IQR)**						
Physical functioning	85(63.8–90)	85(62.5–100)	85(75–100)	95(65–100)		**<0.001**
Role-physical	0(0–100)	100(25–100)	100(50–100)	100(25–100)		**<0.001**
Role-emotional	33.3(0–100)	100(41.7–100)	100(75–100)	100(100–100)		**<0.001**
Vitality	45(35–55)	70(50–70)	65(55–72.5)	60(47.5–80)		**0.003**
Mental health	56(40–76)	76(64–88)	76(58–82)	80(56–88)		**<0.001**
Social functioning	50(25–75)	87.5(62.5–100)	87.5(75–100)	87.5(75–100)		**<0.001**
Bodily pain	35(20–45)	67.5(55–90)	67.5(45–77.5)	67.5(45–90)		**<0.001**
General health	53.3 ± 26.7	67.1 ± 21.9	65.4 ± 21.5	66.9 ± 23.7		**<0.001**
**BAI score, median (IQR)**	9(4–22.5)	6(3–11.5)	5(2–12)	5(1–13.5)		**<0.001**
**BDI score, median (IQR)**	8(3.5–17.5)	4(1–9.5)	3(0–8)	4(0.5–9.5)		**<0.001**
	***Post hoc* comparisons** ^2^					
	**A vs. B**	**A vs. C**	**A vs. D**	**B vs. C**	**B vs. D**	**C vs. D**
**HIT-6 score**	**<0.001**	**<0.001**	**<0.001**	1.00	0.770	1.000
**SF36 HRQoL**						
Physical functioning	0.446	**0.022**	**0.001**	1.00	0.291	1.00
Role-physical	**0.041**	**0.030**	**0.010**	1.00	1.00	1.00
Role-emotional	0.156	**0.030**	**0.012**	1.00	1.00	1.00
Vitality	0.148	**0.019**	**0.011**	1.00	1.00	1.00
Mental health	**0.026**	**0.003**	**0.002**	1.00	1.00	1.00
Social functioning	**0.019**	**0.005**	**0.004**	1.00	1.00	1.00
Bodily pain	**0.001**	**<0.001**	**<0.001**	1.00	1.00	1.00
General health	**0.037**	**0.001**	**0.001**	1.00	1.00	1.00
**BAI**	**0.001**	**<0.001**	**0.001**	0.523	1.00	1.00
**BDI**	**0.048**	**<0.001**	**<0.001**	0.742	0.295	1.00

MIDAS, Migraine Disability Assessment Scale; HIT-6, Headache Impact Test-6; PROM, Patient-reported outcome measures; HRQoL, health-related quality of life; BAI, Beck Anxiety Inventory; BDI, Beck Depression inventory. ^1^Friedman Test, ^2^Bonferroni corrected Wilcoxon Signed Rank test. Values in bold indicate statistical significance (*p* < 0.05).

### 3.5 HRQoL and emotional state

In comparison to baseline values, galcanezumab treatment led to significant improvement in each domain of SF-36 HRQoL at the 2nd month (*p* ranged 0.019 to <0.001) and 3rd month (*p* ranged 0.012 to <0.001). Notably, improvements were observed in all domains, except for physical functioning, role-emotional, and vitality, at the 1st month (Table 4).

From baseline to the 1st, 2nd, and 3rd month visits, the median (IQR) BAI scores showed an important decrease, decreasing from 9 (4–22.5) to 6 (3–11.5), 5 (2-12), and 5 (1–13.5), respectively (*p* < 0.001 for each). Similarly, the BDI scores also significantly decreased from 8 (3.5–17.5) at baseline to 4 (1–9.5), 3 (0–8), and 4 (0.5–9.5) at the 1st, 2nd, and 3rd month visits, respectively (*p* = 0.048, *p* < 0.001, and *p* < 0.001, respectively).

**Figure 2:**
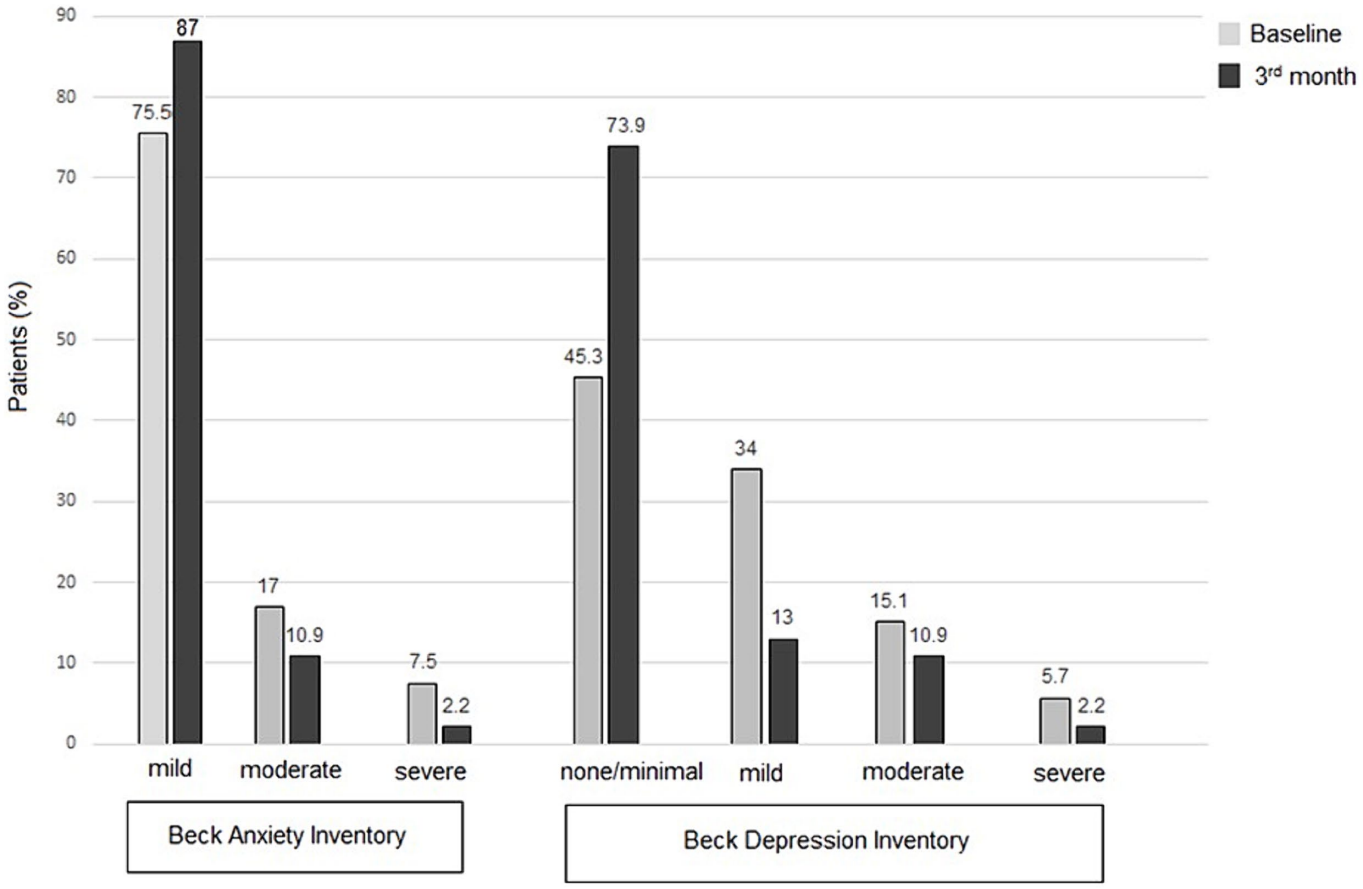
Patient-reported outcome measures for anxiety (BAI scores) and depression (BDI scores), at baseline and follow-up visits.

Notably, BDI scores indicated none or minimal depressive symptoms in 45.3% of patients at baseline, increasing to 77.6, 78.7, and 73.9% at the 1st, 2nd, and 3rd month follow-ups, respectively. Regarding anxiety, 24.5% of patients reported moderate-to-severe symptoms at baseline, which decreased to 10.2%, 8.6%, and 13.0% at the 1st, 2nd, and 3rd month follow-ups, respectively (Table 4; Figure 2).

No meaningful differences were observed between the 1st to 3rd month follow-up visits in terms of HRQoL and emotional state (Table 4).

### 3.6 Response to galcanezumab treatment

Median (IQR) response rates, represented as the percentage reduction in MHDs, were 75% (90–100%), 92.5% (80–100%), and 92.5% (80–100%) at the 1st, 2nd, and 3rd months, respectively. There were 34 patients (63%) in this cohort who showed a 50% or greater reduction in MHDs. Galcanezumab treatment was deemed effective or very effective by the majority of patients and physicians at each visit: 83.6% and 84.1% at the 1st month, 87.7% and 93.4% at the 2nd month, and 85.4% and 89.7% at the 3rd month. Only 2 (4.1%) patients considered galcanezumab not effective at the 1st month, and none of the patients or physicians regarded it as ineffective at the 3rd month.

### 3.7 Safety data

No reportable safety concerns were recorded in our cohort. Considering the TEAEs reported at the 1st, 2nd, and 3rd month visits, constipation was reported in 8 (16.7%), 10 (19.6%), and 9 (17.3%) patients, respectively. Pain at the injection site was noted in 5 (10.4%), 4 (7.8%), and 8 (15.4%) patients, respectively. Nausea was absent in the 1st month, then it was reported by 4 (7.8%) patients in the 2nd month, and by 3 (5.8%) patients in the 3rd month. None of the patients reported elevated blood pressure, diarrhea, or weight loss during the follow-up visits (Table 5).

**Table 5:** Galcanezumab treatment-emergent adverse events.

	After first injection	After second injection	After third injection
**Adverse events, *n*(%)**			
Constipation	8 (16.7)	10(19.6)	9(17.3)
Pain at injection site	5(10.4)	4(7.8)	8(15.4)
Hair loss	1(2.1)	2(3.9)	1(1.9)
Worsening in headache	1 (2.1)	1(2)	1(1.9)
Nausea	0	4(7.8)	3(5.8)
Allergic reaction	0	3(5.9)	2(3.8)
Weight gain	0	2(3.9)	2(3.8)
Cold, flu	0	1(2.0)	1(1.9)
Anxiety, depression	0	1(2.0)	2(3.8)
Generalized muscle ache	0	1(2.0)	1(1.9)
Joint pain	0	0	1(1.9)
Weight loss	0	0	0
Diarrhea	0	0	0
Elevated blood pressure	0	0	0
Other^*^	5(10.4)	7(13.7)	10(19.2)

*n*, Number of subject, ^*^Other: 1st injection: vertigo (2.1%), menstrual changes (2.1%), itching (2.1%), abdominal pain (2.1%), sleep disturbance (2.1%); 2nd injection: menstrual changes (5.9%), vertigo (2.0%), amnesia (2.0%), mood elevation (2.0%), decreased libido (2.0%); 3rd injection: vertigo (5.8%), menstrual changes (3.8%), injection day headache (3.8%), amnesia (1.9%), dizziness (1.9%), sleep disturbance (1.9%).

## 4 Discussion

This real-world study, conducted in patients with EM and CM who had prevalent comorbidities such as sleep disorders and psychiatric problems, demonstrated that galcanezumab treatment was associated with a rapid onset and significant improvement in migraine outcomes. This amelioration included a reduction in MHDs, MMDs, and headache severity. Beyond addressing pain-related parameters, galcanezumab also proved to be effective in enhancing sleep quality in the overall study population, with the decrease in the percentage of patients reporting poor sleep quality (total PSQI scores ≥ 5) by nearly 20% from baseline to the 1st and 2nd months. Besides the overall improvement in sleep quality, galcanezumab specifically improved the total PSQI scores along with the subjective sleep quality and daytime dysfunction domains within a 3-month treatment period in the subgroup of patients with sleep disorder at baseline, and the sleep disturbances domain particularly in patients without baseline depression or anxiety. Moreover, various PROMs, such as HIT-6, MIDAS, and SF-36 HRQoL, as well as BDI and BAI, were rapidly improved following the series of galcanezumab injections. These findings collectively highlight the multifaceted positive impact of galcanezumab on both migraine-related and psychological aspects, emphasizing its potential as an effective therapeutic option in individuals with comorbidities in a real-world clinical setting.

The effect of galcanezumab on sleep parameters in our migraineurs appeared to be a comorbidity-based and domain-specific effect, which involved sleep disturbances domain in patients without depression or anxiety at baseline but the total PSQI scores, subjective sleep quality and daytime dysfunction in those with sleep disorder at baseline.

It is worth noting that, in another real-world study involving galcanezumab in patients with EM and CM, no change in sleep quality, as assessed by the Medical Outcomes Study sleep scale, was observed from baseline after the third and sixth administrations (18). These findings underscore the potential variability in treatment responses and outcomes across different populations and measurement scales in real-world settings.

In a study involving patients with EM and CM receiving erenumab, fremanezumab, or galcanezumab, the PSQI showed a significant reduction from baseline to the 3^rd^ month. However, it did not reach the cut-off of <5 for good sleep quality (32). The study suggests that a more prolonged treatment duration of 6–12 months with erenumab, fremanezumab, or galcanezumab may be required for substantial improvement in sleep quality, especially in CM patients. Additionally, the use of objective measures of sleep quality, such as polysomnography, may reveal positive outcomes even after 3 months of treatment (33, 34). These findings imply the potential for galcanezumab to improve sleep abnormalities over longer treatment periods. The suggestion for further investigation based on objective measures of sleep quality, such as polysomnography, indicates the need for more comprehensive and precise data on sleep parameters in patients undergoing preventive migraine treatments (18).

Although the precise mechanisms are still unclear, CGRP seems to have a role in regulating sleep and arousal. Glutamatergic neurons in the external lateral parabrachial nucleus of the mice, particularly those expressing CGRP, may play a vital role in the arousal response to elevated CO2 or hypoxia. Selective inhibition of these neurons stops waking up in response to CO2 (35). In mouse models with neuropathic pain, preventing sleep fragmentation was achieved by genetically silencing peripheral sensory neurons or ablating CGRP-positive neurons in the parabrachial nucleus (36). CGRP impacts pathways to thalamic trigeminovascular neurons, possibly affecting pain sensitivity in primary headaches during conditions like sleep deprivation (37).

The improvements in sleep parameters (total scores, subjective sleep quality and daytime dysfunction) in the subgroup of patients with baseline sleep disorder is notable given that this group accounts for 40% of the overall study population. The improvement in daytime dysfunction seems to be particularly important since the excessive daytime sleepiness was considered to have a stronger association with the migraine-related disability, compared to other sleep disturbances (i.e., deteriorated sleep quality or increased sleep apnea risk) in patients with CM (38).

Another important finding of the present study seems to be the marked improvement in sleep disturbances domain from baseline to the 3rd month in subgroups of patients with low anxiety and none/minimal depression at baseline. These findings seem notable given the complex association of sleep disturbance with depression, which may precede or follow the onset and recurrence of depression, and the likelihood of individuals with depressive symptoms to suffer from a greater burden of comorbid anxiety symptoms in case of comorbid sleep disturbance (39). Indeed, the sleep disturbance is considered an acute headache trigger for migraine and an independent risk factor for progression from episodic to chronic headache (40). Hence, our findings emphasize that migraine patients without anxiety and/or depression may effectively benefit from galcanezumab, particularly in terms of improving sleep problems through the amelioration of migraine outcomes. However, in those with comorbid anxiety and depression, the improved migraine outcome alone, without addressing the management of psychiatric disorders, may not be sufficient to effectively improve sleep problems.

Some studies reported the association of CM, compared to EM, with higher PSQI scores (worse subjective sleep quality) and higher prevalence of excessive daytime sleepiness and depressive and anxiety symptoms (13, 41, 42). Notably, while our CM and EM patients had similar change from baseline to 3rd month for PSQI total and domain scores, the sleep disturbances domain was particularly improved after galcanezumab treatment in subgroups of patients with low anxiety and none/minimal depression at baseline. Hence, while galcanezumab was effective in ameliorating depressive and anxiety symptoms, its potential to improve sleep disturbances seems to be more prominent in patients without depression or anxiety at baseline. These findings seem to support that in some migraineurs, there is no reciprocal association between negative emotional states and poor sleep quality (43–45).

The observed amelioration in MHDs and MMDs in our current study aligns with other real-world studies on galcanezumab, indicating a more extensive improvement in MHDs and MMDs than reported in randomized controlled trials RCTs (18, 46–48). Notably, in both CM and EM patients, the correlation between MMDs and scores on the MIDAS and the HIT-6 was reported to be stronger during galcanezumab treatment than their correlation recorded at baseline. This emphasizes the presence of treatment benefits extending beyond headache frequency to encompass more subtle aspects of the disease (46, 49, 50).

In our patient population, significant improvements were not only observed in migraine outcome parameters but also in several PROMs linked to migraine-related impairment in functioning. These include substantial improvements in HIT-6 and MIDAS scores, in addition to critical enhancements in SF-36 HRQoL scores. Furthermore, there was a noteworthy enhancement in all domains of HRQoL measured by the SF-36 in galcanezumab-treated patients with both EM and CM. These findings suggest the potential of galcanezumab to alleviate the existing disease burden and improve HRQoL in migraine patients, with implications for increased capabilities in work and daily activities, heightened productivity, and enhanced emotional well-being (51, 52).

Other real-world studies also indicated that monthly prophylactic treatment with galcanezumab was effective in both CM and EM, especially in reducing migraine burden and disability with significant improvements in several PROMs, including HIT-6, MIDAS, and MSQ (18, 46). In this study, the median MIDAS scores started at 50.0 at baseline, then decreased to 9.0 by the 3rd month, indicating a shift from “severe disability” to “little or no/mild” disability. Also, median HIT-6 scores were 67.0 at baseline and ranged from 56 to 58 after galcanezumab treatment, suggesting amelioration from “severe impact” to “substantial impact.” Nonetheless, while the three-month follow-up provides initial insights, extending this to 6–12 months could offer a better understanding of the long-term effects and sustainability of treatment benefits.

PROMs, reflecting the patient’s perspective and experience, are increasingly used in clinical practice to improve patient-centered care, patient engagement, and shared decision-making (53). Nonetheless, while the disability assessment tools widely used in headache research such as MIDAS and HIT-6 are useful as outcome measures, individually they cannot capture the entire experience of headache disability (54). Also, PROMs measures differ with respect to their ability to capture treatment efficacy from a patient’s perspective and to reliably indicate a patient’s real clinical improvement (54, 55). In this regard, the use of multimodal PROMs that assess migraine as well as comorbidities and QoL in our study seems to strengthen our findings, enabling a concomitant evaluation of several individual variables and a more comprehensive picture of treatment outcomes related to improvement in several domains besides the headache (54, 55).

Disability in migraine patients is a multifaceted phenomenon influenced by personal functioning and the psychological burden of the disease, in addition to the number of headache days (46). In our patient cohort, BDI and BAI scores showed significant improvement following the galcanezumab loading dose, and this effect was sustained throughout the subsequent injection series. Similarly, in another real-world study involving a cohort of 43 patients with HFEM and CM, galcanezumab treatment was associated with improved Migraine-Specific Quality of Life scores and a reduction in depressive symptoms and anxiety (18). The ability of galcanezumab to rapidly alleviate depressive and anxious symptoms is particularly notable, considering that both depression and anxiety are recognized as risk factors for migraine chronification, associated with decreased treatment response, impaired quality of life, and increased overall disease burden (18, 43, 56).

It’s worth noting that almost ~80% of our patients had comorbid sleep disorders or psychiatric diseases before galcanezumab treatment. This suggests that galcanezumab may be a favorable therapeutic option in migraine patients with a considerable burden of comorbidities. In a real-world study with CM patients, the response rate to galcanezumab was 64.3%, with daily headache, the presence of depression, and absence of accompanying symptoms of migraine identified as significant predictors of a poor response to galcanezumab treatment (57). In a *post hoc* analysis of the REGAIN and pooled EVOLVE-1 and EVOLVE-2 studies, a medical history of anxiety and/or depression was reported to interfere with the response to galcanezumab in patients with CM, decreasing the likelihood of a reduction in overall MHD and functional improvement in those with comorbid anxiety and/or depression (58).

The majority of our patients and physicians considered galcanezumab to be effective or very effective at each visit, and the reported TEAEs in our cohort were consistent with the well-known high safety and tolerability profile of galcanezumab (16, 18). In this context, our findings support the notion that the high tolerability of galcanezumab, facilitated by its monthly administration and sustained effectiveness, establishes a significant foundation for improved adherence and, ultimately, enhanced outcomes in patients with both EM and CM (52, 59).

This study has several limitations that should be acknowledged. Firstly, the single-center design with a limited number of participants may raise concerns about the generalizability of the findings, potentially limiting the external validity. Secondly, the analysis of data on negative emotional states and sleep quality relied on self-reported measures using PROMs rather than face-to-face psychiatric evaluations. This method may not fully capture the complexity of emotional and sleep-related conditions. Thirdly, the relatively short 3-month follow-up period might have limitations in capturing the long-term response to galcanezumab and monitoring potential risks associated with the treatment. An extended follow-up duration could provide a more comprehensive understanding of the treatment’s efficacy and safety profile over time. The association of continued treatment beyond 3 months with a likely delayed response in non-responders is recognized in the literature (43). Fourthly, absence of a control group in this real-life data is another limitation, given that inclusion of a control group for comparison would strengthen the conclusions drawn about galcanezumab’s effectiveness against standard care or placebo.

## 5 Conclusion

In conclusion, this real-world study indicates the likelihood of galcanezumab to be a promising and effective emerging agent for migraine prophylaxis, offering not only reduced headache days but also reduced migraine disability and improved functionality and negative emotional states. Galcanezumab demonstrates notable benefits in improving sleep quality, along with a comorbidity-based and domain-specific effect on sleep parameters, which involved sleep disturbances domain in patients without depression or anxiety at baseline but the total PSQI scores, subjective sleep quality and daytime dysfunction in those with sleep disorder at baseline. Given the improved sleep parameters in galcanezumab-treated CM and EM patients within 3-months treatment, the real potential of galcanezumab on improved sleep problems may appear in the longer term. Nonetheless, its effectiveness in population of migraineurs suffering from either comorbid sleep disorder or psychiatric disease seem to indicate the likelihood of galcanezumab to be a favorable therapeutic option in migraine patients with comorbidities. There is a need for real-world studies with longer follow-up periods assessing galcanezumab’s effectiveness against standard care or placebo to better understand effectiveness and safety profile of galcanezumab and to optimize the positioning of this new drug within the current migraine prophylaxis practice.

## Data availability statement

The original contributions presented in the study are included in the article/supplementary material, further inquiries can be directed to the corresponding author.

## Ethics statement

This study was conducted in accordance with the ethical principles stated in the “Declaration of Helsinki” and approved by Acibadem University School of Medicine Medical Research Ethics Committee (Approval number: 2023-20/671). The participants provided their written informed consent to participate in this study.

## Author contributions

EI: Conceptualization, Data curation, Formal analysis, Investigation, Methodology, Project administration, Writing – original draft, Writing – review & editing. TE: Data curation, Formal analysis, Investigation, Writing – review & editing. PY: Data curation, Formal analysis, Investigation, Supervision, Writing – review & editing.
